# Response of Bone Resorption Markers to *Aristolochia longa* Intake by Algerian Breast Cancer Postmenopausal Women

**DOI:** 10.1155/2014/820589

**Published:** 2014-04-30

**Authors:** Bachir Benarba, Boumedienne Meddah, Aicha Tir Touil

**Affiliations:** ^1^Laboratory of Bioconversion, Microbial engineering and Health Safety, Department of Biology, University of Mascara, 29000 Mascara, Algeria; ^2^Laboratory of Research on Biological Systems and Geomatics, University of Mascara, 29000 Mascara, Algeria

## Abstract

*Aristolochia longa* is widely used in traditional medicine in Algeria to treat breast cancer. The aim of the present study was to investigate the response of bone resorption markers to *A. longa* intake by Algerian breast cancer postmenopausal women. According to the *A. longa* intake, breast cancer patients were grouped into *A. longa* group (Al) (*n* = 54) and non-*A. longa* group (non-Al) (*n* = 24). 32 women constituted the control group. Bone resorption markers (from urine) pyridinoline (PYD) and deoxypyridinoline (DPD) were determined by HPLC. Serum and urinary creatinine, uric acid, and urea were measured. 1 g of *A. longa* intake resulted in significant rise of renal serum markers and a pronounced increase of bone resorption markers. The intake of *A. longa* roots is detrimental for kidney function and resulted in high bone resorption, maybe due to the reduction in renal function caused by the aristolochic acids contained in the roots.

## 1. Introduction

**Figure 1:**
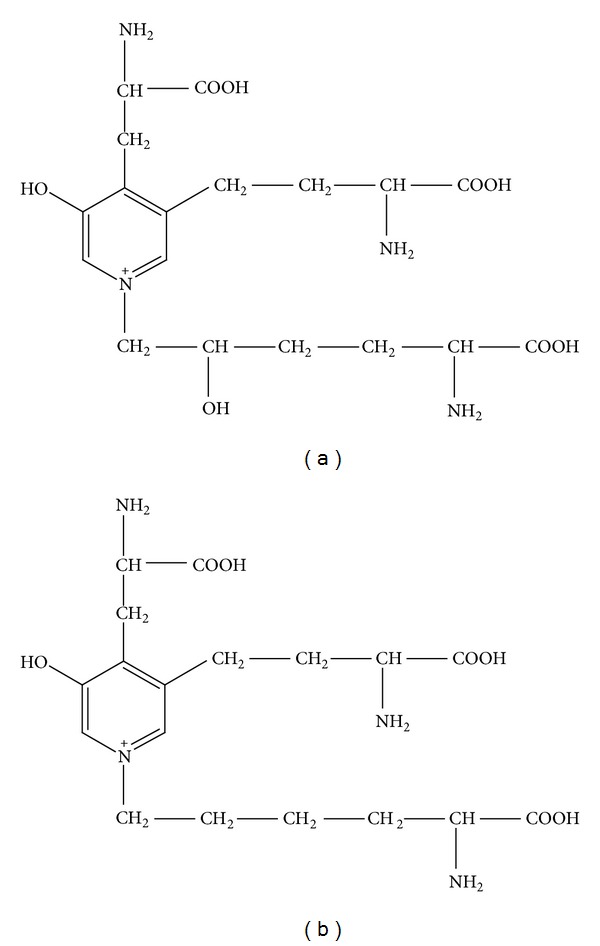
Structures of pyridinoline (PYD) and deoxypyridinoline (DPD). Upper: pyridinoline (molecular weight: 429); lower, deoxypyridinoline (molecular weight: 413).

The pyridinium cross-links pyridinoline (PYD) and deoxypyridinoline (DPD) are established markers of bone resorption measured in blood and urine and are used to investigate bone metabolism and manage bone diseases [1]. Deoxypyridinoline (Dpd) distributed mostly in bone collagen has a higher specificity for bone than pyridinoline (pyd), which is excreted in urine, and it is not affected by diet, whereas pyridinoline (Pyd) is abundant in bone and cartilage (Figure 1) [2]. They are inexpensive, sensitive, and useful for the diagnosis of bone metastasis [3].

*Aristolochia longa* belongs to the genus* Aristolochia* (Aristolochiaceae) consisting of about 500 species mostly distributed along tropical, subtropical, and Mediterranean regions of the world [4]. The plant is widely used in traditional medicine in Algeria [5]. Although plants of the genus* Aristolochia *have been shown to exhibit interesting anticancer activities including cytotoxic and apoptosis-induced, herbal remedies containing plants of* Aristolochia* genus are banned in many countries because of the nephrotoxicity of their aristolochic acid [6, 7].

The aim of the present study was to investigate the response of bone resorption markers to* A. longa *intake by Algerian breast cancer postmenopausal women.

## 2. Materials and Methods

### 2.1. Subjects

A total of 110 postmenopausal women were recruited into the study from two hospitals in the State of Mascara (north-west of Algeria). Of these, 32 women (age: 66.4 ± 10.45 years) free from any critical illness or medical problems, constituted the control group and 78 were newly diagnosed with primary breast cancer. Patients with recurrent breast cancer, adjuvant therapy (radiotherapy or chemotherapy) prior to surgery, or a history of previous cancer, renal failure, osteoporosis, connective tissue disease, degenerative bone disease traumatic fracture, early menopause before 40 years of age, and current medication use that may affect bone density, including hormones, vitamins, and mineral supplements, were excluded from the study.

*Aristolochia longa* intake was assessed retrospectively using a standardized questionnaire. Subjects were interviewed after diagnosis and before initiating treatment. Breast cancer patients were then grouped into* A. longa* group (Al) (*n* = 54) andnon-*A. longa* group (non-Al) (*n* = 24) (Table 1).

### 2.2. Specimen Collection

Serum samples separated from antecubital venous blood were centrifuged (1500 g/minute, for 10 minutes). Fresh urine samples were taken at 8–10 AM. Specimens were stored at −20°C until the time of analysis.

### 2.3. Analysis of Bone Markers

Pyridinoline (PYD) and deoxypyridinoline (DPD) were determined by HPLC. Total urinary PYD and D-PYD were determined as described [8]. In brief, 1 mL urine was hydrolyzed with an equal volume of 12 mol/L HCl at 105°C for 16 h to convert all urinary crosslinks to the peptide-free form. Free urinary PYD and DPD were determined without hydrolysis. After partition chromatography on CFl cellulose, samples and external standards were separated by high performance liquid chromatography (HPLC), and concentrations were determined by fluorometry of the eluent peaks. Standards were derived from standard solution with 249 nmol of PYD and 30 nmol of DPD (a generous gift from Pr Brazier, Clinic pharmacology Lab, University of Amiens, France). To correct for variations in urinary flow, PYD and DPD results were normalized to the urinary creatinine concentration and expressed as nanomoles (PYD, DPD) divided by millimoles creatinine (nM (PYD, DPD)/mM creatinine). Urinary creatinine was measured according to Jaffe method (Fluitest Crea, Biocon Diagnostic, Ref 448, Lot G558, Denmark).

### 2.4. Statistics

All numeric data were expressed as mean ±SD. Data were analyzed using the student *t*-test. A *P* value ≤0.05 was considered significant. Statistical calculations were performed with SPSS version 9.0.

## 3. Results

One hundred and sixteen women newly diagnosed with primary breast cancer were initially included, of whom 92 women (80%) had used local medicinal plants three months before diagnosis. For 81 women of them, the used plants were known. 24 women did never use medicinal plants. 54 women had consumed 1 ± 0.3 g (0.85–1.5 g)/day/40 days of* A. longa* roots. There was no statistical difference between the three groups (A, B, and control) with regard to age, BMI, and time after menopause. Age, time after menopause, and BMI are shown in Table 1.

According to our results (Table 2), 1 g of* A. longa *intake resulted in significant rise of renal serum markers. In the breast cancer population, mean serum creatinine, urea, and uric acid were significantly higher in patients of Al group than those of non-Al group by 36.63%, 68.36%, and 73.96%, respectively. When compared to controls, they had significantly higher levels of serum creatinine, urea, and uric acid (1.38 ± 0.17 versus 0.93 ± 0.1, 42.26 ± 8.13 versus 23.89 ± 2.66, and 07.15 ± 1.89 versus 03.87 ± 0.42, resp.).

Patients newly diagnosed for breast cancer (Al and non-Al patients) had significantly higher concentrations of DPD and PYD (all forms) than controls. Interestingly, the intake of 1 g of* A. longa *by breast cancer patients resulted in a pronounced significant increase of mean concentrations of bone resorption markers. Mean concentrations of DPD (free, conjugated, and total form) increased by 71.55%, 20.85%, and 54.08%, respectively. On the other hand, median concentrations of PYD (free, conjugated, and total form) were significantly higher by 83.26%, 34.22%, and 19.06%, respectively (Table 3).

## 4. Discussion

*A. longa* is widely used in anticancer treatment in Algeria [9] and Morocco [10]. We have recently demonstrated that an aqueous extract of* A. longa* induced cell death of Burkitt's lymphoma cell line (BL41) in a dose-dependent manner. The IC_50_ of* A. longa* aqueous extract was estimated at about 15,63 *μ*g/mL. The extract induced apoptosis in Burkitt's lymphoma BL41 cells, by triggering the mitochondrial pathway (disruption of ΔΨm, activation of caspases 9 and 3, and PARP cleavage) [11]. To our knowledge, the present study is the first to investigate the effect of* A. longa* intake by breast cancer patients on bone resorption.

Our results showed that intake of 1 g of* A. longa *resulted in marked increasing of serum creatinine, urea, and uric acid levels. Here we give evidence that* A. longa *may be detrimental for kidney function in breast cancer patients. Indeed, many* Aristolochia* plants were reported to cause nephropathy [12, 13]. Our findings support the association established between ingestion of herbal remedies containing aristolochic acids (AAs) and the development of a renal disease, designated as aristolochic acid nephropathy (AAN) [14]. Shibutani et al. [15] concluded that AA-I is solely responsible for the nephrotoxicity associated with AAN, since kidney is the primary target organ for its toxicity. Recently, Cherif et al. [5] isolated and characterized AAI from* A. longa* growing in Algeria. The mechanisms by which AA induces renal interstitial fibrosis are still largely unknown, but defective activation of antioxidative enzymes and mitochondrial damage caused by AA tubular toxicity, impaired regeneration of proximal tubular epithelial cells, and apoptosis secondary to caspase-3 activation may be involved [16].

Measurement of the metabolites of type I collagen, the predominant collagen in bone, like PYD and DPD, has been reported to be useful for monitoring bone turnover in many different disorders, including diseases with bone metastases [17]. Findings of the present study showed that breast cancer patients with 1 g* A. longa *intake were characterized by high bone resorption as assessed by PYD and DPD. Since breast cancer patients included in the present study were newly diagnosed with breast cancer without any chemotherapy, the increased bone resorption may be due to secondary causes of bone loss unrelated to cancer treatment. Secondary causes of bone loss are factors other than menopause and aging that can lead to osteoporosis [18]. Kidney failure is one of the secondary causes of osteoporosis in postmenopausal women. Klawansky et al. [19] found that 85% of women with osteoporosis have some degree of renal compromise. We suggest that in postmenopausal women newly diagnosed with breast cancer, the intake of 1 g of* A. longa* roots could contribute to bone loss, maybe due to the reduction in renal function caused by the aristolochic acids contained in the roots. Our hypothesis may be supported by the relationship demonstrated in many studies between reduction in renal function and increased bone loss [20, 21].

Our study has several limitations. Bone formation markers were not measured. Serum vitamin D and PTH levels which are important mediators in the association between renal function and bone were not assessed.

## 5. Conclusion

In postmenopausal women newly diagnosed for breast cancer, the intake of* A. longa* roots is detrimental for kidney function and resulted in high bone resorption, maybe due to the reduction in renal function caused by the aristolochic acids contained in the roots.

## Figures and Tables

**Table 1:** Age, BMI, and time after menopause of the study population.

	Control (*n* = 32)	Al (*n* = 54)	Non-Al (*n* = 24)
Age (yrs)	66.4 ± 10.45 (49–78)	65.05 ± 10.02 (50–77)	65.87 ± 08.64 (52–80)
BMI (kg/m^2^)	24.09 ± 04.32 (21.3–28)	25.21 ± 03.89 (23.43–28.2)	24.77 ± 06.02 (21–27)
Time after menopause (yrs)	15.09 ± 04.11 (5–21)	15.83 ± 05.46 (7–29)	14.98 ± 14 (4–27)

**Table 2:** Serum creatinine, BUN, and uric acid.

	Control (*n* = 32)	Al (*n* = 54)	Non-Al (*n* = 24)
Creatinine (mg/dL)	0.93 ± 0.1	1.38 ± 0.17^a,b^	1.01 ± 0.09
BUN (mg/dL)	23.89 ± 2.66	42.26 ± 8.13^a,b^	25.10 ± 5.78
Uric acid (mg/dL)	03.87 ± 0.42	07.15 ± 01.89^a,b^	04.11 ± 0.81

^a^High significant value (*P* < 0.01) as compared to controls.

^b^High significant value (*P* < 0.01) as compared to non-Al group.

**Table 3:** Bone resorption markers PYD and DPD concentrations.

	PYD total form (nmol/mmol Cr)	PYD-free form (nmol/mmol Cr)	PYD conjugated form (nmol/mmol Cr)	DPD total form (nmol/mmol Cr)	DPD-free form (nmol/mmol Cr)	DPD conjugated form (nmol/mmol Cr)
Controls	41.2 ± 9.2	32.14 ± 10.4	10.16 ± 8.9	18.9 ± 10.2	8.2 ± 3.11	11.4 ± 6.3
Al	82.63 ± 19.33^a,b^	71.86 ± 15.77^a,b^	25.02 ± 08.47^a,b^	46.55 ± 11.52^a,b^	23.04 ± 04.87^a,b^	30.71 ± 06.89^a,b^
Non-Al	69.40 ± 12.17^a^	39.21 ± 14.92^a^	18.64 ± 05.82^a^	30.21 ± 09.25^a^	13.43 ± 07.76^a^	25.41 ± 11.35^a^

^a^High significant value (*P* < 0.01) as compared to controls.

^b^High significant value (*P* < 0.01) as compared to non-Al group.
